# Physical Therapists’ Acceptance of a Wearable, Fabric-Based Sensor System (Motion Tape) for Use in Clinical Practice: Qualitative Focus Group Study

**DOI:** 10.2196/55246

**Published:** 2024-02-29

**Authors:** Audrey Lee, Patricia Dionicio, Emilia Farcas, Job Godino, Kevin Patrick, Elijah Wyckoff, Kenneth J Loh, Sara Gombatto

**Affiliations:** 1 Department of Bioengineering San Diego State University San Diego, CA United States; 2 Joint Doctoral Program in Public Health San Diego State University and University of California San Diego San Diego, CA United States; 3 Qualcomm Institute University of California San Diego La Jolla, CA United States; 4 School of Public Health University of California San Diego La Jolla, CA United States; 5 Active, Responsive, Multifunctional, and Ordered-materials Research (ARMOR) Laboratory Department of Structural Engineering University of California San Diego La Jolla, CA United States; 6 School of Exercise & Nutritional Sciences College of Health & Human Services San Diego State University San Diego, CA United States

**Keywords:** low back pain, physical therapy, physical therapist, wearable sensor, technology acceptance model, motion tape, kinesiology tape

## Abstract

**Background:**

Low back pain (LBP) is a costly global health condition that affects individuals of all ages and genders. Physical therapy (PT) is a commonly used and effective intervention for the management of LBP and incorporates movement assessment and therapeutic exercise. A newly developed wearable, fabric-based sensor system, Motion Tape, uses novel sensing and data modeling to measure lumbar spine movements unobtrusively and thus offers potential benefits when used in conjunction with PT. However, physical therapists’ acceptance of Motion Tape remains unexplored.

**Objective:**

The primary aim of this research study was to evaluate physical therapists’ acceptance of Motion Tape to be used for the management of LBP. The secondary aim was to explore physical therapists’ recommendations for future device development.

**Methods:**

Licensed physical therapists from the American Physical Therapy Association Academy of Leadership Technology Special Interest Group participated in this study. Overall, 2 focus groups (FGs; N=8) were conducted, in which participants were presented with Motion Tape samples and examples of app data output on a poster. Informed by the Technology Acceptance Model, we conducted semistructured FGs and explored the wearability, usefulness, and ease of use of and suggestions for improvements in Motion Tape for PT management of LBP. FG data were transcribed and analyzed using rapid qualitative analysis.

**Results:**

Regarding wearability, participants perceived that Motion Tape would be able to adhere for several days, with some variability owing to external factors. Feedback was positive for the low-profile and universal fit, but discomfort owing to wires and potential friction with clothing was of concern. Other concerns included difficulty with self-application and potential skin sensitivity. Regarding usefulness, participants expressed that Motion Tape would enhance the efficiency and specificity of assessments and treatment. Regarding ease of use, participants stated that the app would be easy, but data management and challenges with interpretation were of concern. Physical therapists provided several recommendations for future design improvements including having a wireless system or removable wires, customizable sizes for the tape, and output including range of motion data and summary graphs and adding app features that consider patient input and context.

**Conclusions:**

Several themes related to Motion Tape’s wearability, usefulness, and ease of use were identified. Overall, physical therapists expressed acceptance of Motion Tape’s potential for assessing and monitoring low back posture and movement, both within and outside clinical settings. Participants expressed that Motion Tape would be a valuable tool for the personalized treatment of LBP but highlighted several future improvements needed for Motion Tape to be used in practice.

## Introduction

### Prevalence and Impact of Low Back Pain

Low back pain (LBP) is one of the world’s leading causes of disability [1-3]. In 2019, there were approximately 568.4 million prevalent cases, 223.5 million incident cases, and 63.7 million cases of years lived with disability owing to LBP reported globally [4]. LBP affects all ages and genders, but its prevalence increases with age, peaking at the age of approximately 45 to 54 years [4]. Approximately 70% to 85% of adults are expected to experience at least 1 episode of LBP in their lifetime [5]. Once predisposed to LBP, individuals are twice as likely to experience recurrent episodes of LBP [6]. Annually, LBP in the United States results in 149 million missed work days [7]. The total costs of LBP worldwide amount to approximately US $100 billion a year, with two-thirds of this amount owing to lost wages and decreased work productivity [8].

### Treatment of LBP With Physical Therapy

Physical therapy (PT) is a common, effective, evidence-based treatment for LBP [9,10]. Specifically, active interventions including exercises prescribed by a physical therapist are effective for prevention and treatment of LBP [11,12]. During an initial examination, a physical therapist can identify musculoskeletal and neuromuscular impairments associated with the LBP problem by conducting assessment of the patient’s posture and movement. Then, the physical therapist and patient can work together to promote strength, stability, and mobility with in-clinic sessions and an assigned home exercise program with the goal of decreasing pain and disability [10,13]. Monitoring the patient’s posture and movement can provide a basis for determining individualized factors associated with the LBP problem, which can then be addressed through targeted interventions.

### Incorporation of Technology in PT

Whether at home or at work, specific movement patterns that are performed repeatedly have been identified as a significant risk factor for the development and persistence of LBP [2,14,15]. These movement patterns of the low back region can be characterized by evaluating the angle, velocity, and acceleration [16] and can assist in LBP diagnosis, treatment, and prevention. There are several approaches to monitoring spine posture and movement. Generally, when conducting a PT examination, clinicians visually monitor posture and movement or use tools that measure the range of movement such as goniometers or inclinometers [17], but an alternative approach is to use technology to help better quantify the objective measures of spine posture and movement and offer potential benefits such as remote monitoring [16,18,19] while the patient is away from the clinic.

### Technologies for Monitoring LBP

To date, existing technologies used to measure spine posture and movement in research and practice include optical motion capture, inertial measurement units (IMUs), and other wearable sensors [20-22]. Despite the variety of systems available, they generally present ≥1 limitation. Optical motion capture systems offer great precision and accuracy in monitoring human movement. However, their applications are limited owing to space needs, cost, and level of expertise needed. IMUs are portable devices that measure metrics such as acceleration and orientation [23] and include a variety of wearable sensors such as accelerometers, gyroscopes, and magnetometers, making them ideal for collecting data in a free-living environment. However, when used for monitoring human movement, IMUs have several limitations including decreased accuracy and precision for measuring slow movements [24,25], difficulty with measuring the axial plane movement accurately, inability to account for the multisegmented nature of the spine [26], and the need for multiple IMUs to triangulate posture and movement of a segment that can be cumbersome to the wearer [27].

### Motion Tape

Owing to the limitations of existing sensor systems for measuring spine posture and movement, there is a need to explore new sensor innovations to address this issue. Ideally, such an approach would be wearable, unobtrusive, and usable in a clinical environment during PT sessions and in a person’s natural environment to support home-based care. Another desired requirement would be high accuracy while collecting posture and movement data for a prolonged period.

Motion Tape, developed by Loh and Lin [28], is a disposable, self-adhesive skin-strain sensor system made using graphene nanosheets coated onto commercially available kinesiology tape (also known as K-Tape) [29-33]. Motion Tape has piezoresistive properties based on the deformation of the integrated graphene nanosheets in the tape that makes it sensitive to strain [33]. In previous studies, Motion Tape has demonstrated stable performance under cyclic strains [33,34]. In addition, the Motion Tape sensor system has been tested on human participants [33,34], displaying accuracy in measuring skin strains and angles across biceps, knees, shoulders, wrists, and various other body regions when compared with IMUs and skin strains estimated using optical motion capture systems [35]. Overall, Motion Tape offers noninvasive, comfortable, and practical skin-strain measurements and can comprehensively capture complex movements and muscle engagement, especially when applied as a network of sensors [35].

### Motion Tape for a Low Back Use Case

When used for a low back use case, Motion Tape provides a means to capture the lumbar spine’s multisegmental nature and multiplanar movements [36]. Motion Tape’s low-profile and stretchable nature allows it to be worn throughout the day for all human shapes and sizes, and it could be suitable for use in an individual’s natural environment with minimal interference to their daily activities. Motion Tape provides unique sensing streams that can be used in machine learning and artificial intelligence models to optimize inferences related to the management of LBP. Specifically, Motion Tape for a low back use case can address several key issues in a physical therapist’s management of LBP, including the following: expanding on the level of detail available during the clinical assessment of posture and movement, assessing spinal posture and movement in a free-living environment, use for the promotion of engagement and adherence with and precise performance of a prescribed home exercise program, and using the patient’s response to treatment to make informed decisions for future treatment or other patients [37]. Although there are several potential benefits that Motion Tape may add to personalized health care for LBP, the acceptability of Motion Tape among physical therapists has yet to be assessed.

### Physical Therapists’ Acceptance of Motion Tape

The success of this device is dependent on user acceptance or one’s belief that the device will help them perform their work better (ie, perceived usefulness) and that the device’s performance benefits outweigh the effort of using the device (ie, perceived ease of use) [38]. Thus, it is vital to understand physical therapists’ perspectives about Motion Tape and their willingness to use it in their practice, to inform future developments and improvement of the technology.

### Problem Statement

The primary aim of this research study was to evaluate physical therapists’ acceptance of Motion Tape for the management of LBP. The secondary purpose was to explore physical therapists’ current needs and recommendations regarding future development of Motion Tape.

## Methods

### Device Description and Stage of Development

**Figure 1:**
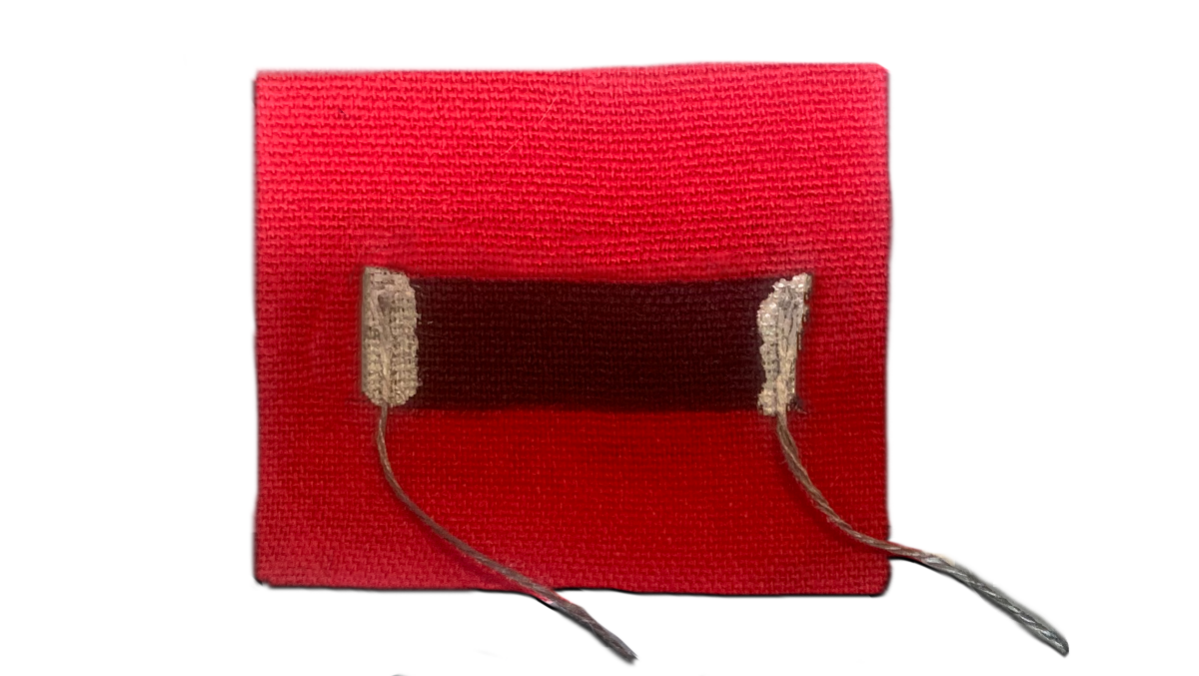
Motion Tape sample with conductive wire leads given to the physical therapists for evaluation.

In this study, licensed physical therapists evaluated a prototype of Motion Tape and examples of data streams from the app for a low back use case. The Motion Tape samples evaluated in this study included the Motion Tape sensor system with conductive wire leads connected to both sides of the sample (Figure 1).

### Study Design

**Figure 2:**
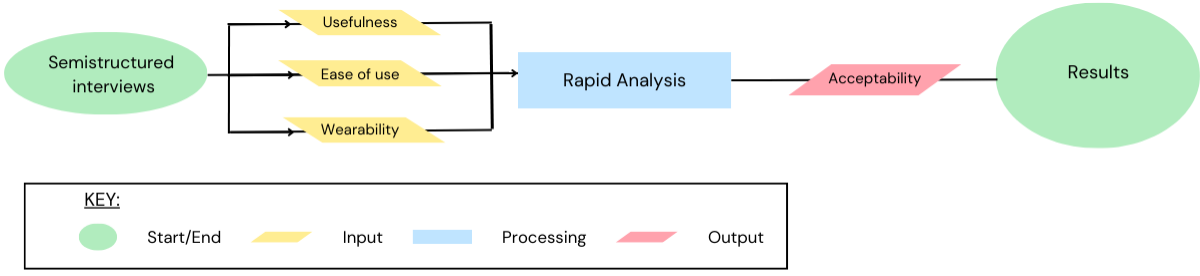
Study design overview—the evaluation of Motion Tape’s acceptability.

This exploratory, qualitative study was designed to explore physical therapists’ acceptance of Motion Tape to provide a basis for future device development (Figure 2). The study was conducted from a constructivist point of view, with the goal of gaining insightful accounts and narrations of clinicians’ lived experiences with technology and patients, rather than identifying an absolute truth [39]. We used semistructured focus groups (FGs) that incorporated human factor considerations to uncover real-world needs and obstacles and to ensure that the development of the sensor system can be informed by real-world PT clinical needs.

### Theoretical Framework and Constructs

The Technology Acceptance Model (TAM) framework was used in this study to assess two determinants of user acceptance of or willingness to use a technology: (1) perceived usefulness and (2) perceived ease of use [38,40]. An additional factor of wearability was also assessed to examine physical therapists’ perceptions about patient-centered issues that would affect whether the device would be worn [41]. Recommendations for future improvements were also investigated to collect insight into data, device, and app developments that clinicians would like to see for Motion Tape.

Perceived usefulness was defined as the degree to which the use of Motion Tape would enhance the physical therapists’ management of LBP [39-42], and this was assessed using the following constructs: (1) productivity, (2) effectiveness, (3) ability to make their job easy, and (4) benefits to PT treatment and recovery. Perceived ease of use was defined as the degree to which the use of Motion Tape would be effortless when used for managing LBP [39-42], and this was assessed using the following constructs: (1) how easy it would be for physical therapists to learn how to use it, (2) what level of instruction would physical therapists need to use it, and (3) how clear and understandable Motion Tape was in its current state. Wearability was defined as the degree to which Motion Tape would fit well and be comfortable for patients to wear on their back [42], and this was assessed based on (1) adhesion, (2) fit, (3) feel, and (4) how comfortable physical therapists would feel about applying and prescribing Motion Tape.

### Participants and Setting

This study was conducted at the American Physical Therapy Association’s (APTA’s) Combined Sections Meeting (San Diego, California) on February 24, 2023. Participants were recruited by sending study information via email to physical therapists who were members of the APTA Academy of Leadership Technology Special Interest Group. Members were also offered an opportunity to participate when they attended the Technology Special Interest Group in-person meeting at the APTA Combined Sections Meeting. Individuals were included in this study if they were a licensed physical therapist and were excluded from participating if they were unable to respond to questions in English. In total, 8 physical therapists were eligible and agreed to participate in 2 FGs of 4 clinicians each. A sample size of 8 people, in 2 FGs, was considered sufficient for this qualitative study to provide adequate variability and data saturation [43] and to provide a basis for device improvement. In addition, after data from the 2 FGs were collected and analyzed, the data were deemed saturated (ie, no new themes or codes were generated) and no further FGs were needed.

### Ethical Considerations

The study protocol was considered to be exempt from ethics approval by the San Diego State University institutional review board. Each participant provided written consent before participating.

### FG Methods

An FG guide (Multimedia Appendix 1) was used to lead the group’s discussion. The FG guide was developed by investigators (AL, PD, and SG) to be semistructured with open-ended questions to explore the participants’ perspectives about the usefulness, usability, and wearability of Motion Tape and to collect insight into future improvements for the sensors and data visualization (Textbox 1). A template of the FG guide was piloted with a Doctor of Physical Therapy student and a physical therapist at San Diego State University to ensure credibility [44]. General domains for each construct were prespecified to correspond with each interview question. Domains were defined based on the TAM framework and included perceived usefulness and perceived ease of use. An additional domain of wearability also was assessed.

**Guiding questions from the focus group guide.**

**Perceived wearability (W)**

- How secure do you think the Motion Tape adhesive will be? (W-adhesion)
- To what degree do you think these sensors would fit your patients’ anatomy (ie, their low back)? (W-fit)
- To what degree do you think your patients would feel the sensors on their back? (W-feel)
- How do you predict the Motion Tape Sensors would feel when being removed? (W-feel)

**Perceived usefulness (U)**

- To what degree would the usage of Motion Tape sensors affect how quickly you can assess your patient’s posture, movement, or exercise performance? (U-efficiency)
- How effective do you think the Motion Tape sensors will be to capture valid data on your patients in the clinic? (U-effectiveness)
- How effective do you think the Motion Tape will be to capture valid data on your patients in their daily routine and normal environment? (U-effectiveness)
- To what degree would the usage of Motion Tape sensors affect the level of difficulty of your job as a clinician/physical therapist? (U–make job easier)
- What features, if any, would make the Motion Tape more useful to you? (U-useful)

**Perceived ease of use (EU)**

- How easy do you think it would be to learn how to use Motion Tape? (EU-easy to learn)
- How comfortable would you feel prescribing Motion Tape to a patient to monitor their movements at home? (EU-comfort in usage)
- What level of knowledge do you think a clinician/PT would need to use the Motion Tape? (EU-clear and understandable)
- How easy/difficult do you think it would be for a clinician/PT to apply the Motion Tape to the patient's back? (EU-easy to use)
- What features, if any, would make the Motion Tape easier for you to use? (EU-easy to use)

FGs were conducted by AL (a female Master of Science student investigator) and PD (a female PhD student investigator). Reflexivity was maintained by the research team by discussing assumptions and biases that may influence how the clinicians responded to the FG moderators, who were not licensed physical therapists. As SG is a licensed physical therapist and member of APTA, she was able to provide valuable insight during the development of the interview guide, analysis, and interpretation to ensure credibility of the findings [44].

**Figure 3:**
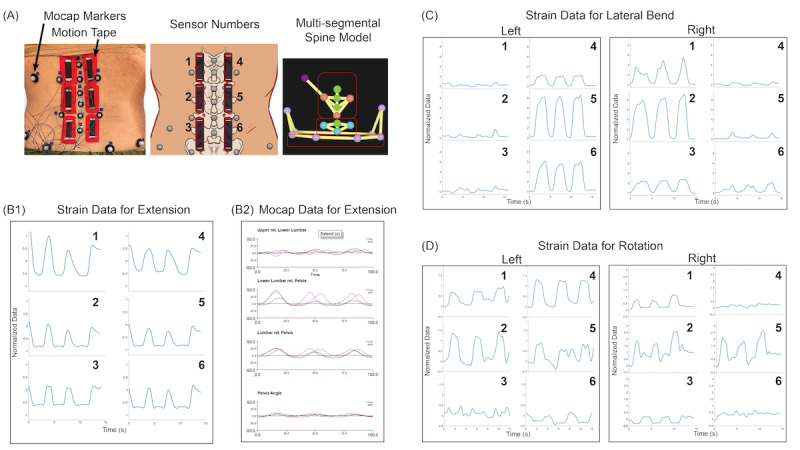
Poster of Motion Tape placement and app data output for a low back use case. (A) The laboratory setup with 6 pieces of Motion Tape and several optical motion capture markers on anatomical landmarks of the lumbar spine. (B) The graphs display the following: (1) blue—the normalized strain data for extension, captured by the 6 Motion Tapes, and (2) purple—the kinematics for extension in degrees, captured by the optical motion capture system (reference standard). (C) The normalized strain data for right and left lateral bending obtained from the 6 Motion Tapes. (D) The normalized strain data for right and left rotation obtained from the 6 Motion Tapes.

FGs were anonymized, and each participant was assigned a color as a name to ensure confidentiality. Each FG lasted approximately 1 hour and was recorded using digital voice recorders (Olympus Voice Recorder; WS-853). Before asking the participants questions, the investigators gave each participant a sample of Motion Tape. Participants were then oriented to a poster that displayed the Motion Tape placement and app data output streams (Figure 3).

### Data Processing and Analysis

All FG audio data were downloaded to a HIPAA (Health Insurance Portability and Accountability Act)-compliant laboratory server, accessible only to the research staff, and removed from the digital voice recorder. The recordings were then transcribed, first using computer-based transcription (Word; Microsoft Corp). An investigator then checked and verified each transcription by listening to the original audio and reviewing and correcting the computer-based transcription.

Considering the need for timely feedback in the sensor development process, we adopted a rapid qualitative analysis (RQA) approach to explore themes regarding the acceptability and wearability of Motion Tape [45]. RQA was conducted by 3 investigators to assess the FG responses effectively and efficiently and to identify major themes. Codes and themes for RQA were deductively developed based on the TAM framework and the study objective [41]. We then used an inductive approach to generate RQA codes and themes, allowing for quick sorting of FG dialogue.

To ensure rigor and consistency of the method, a constant comparative approach with investigator triangulation was used at each stage [46]. First, the 3 investigators independently completed a summary report for each FG, with quotes and relevant topics under the respective themes and codes. Once the individual coding and summary reports for both FGs were completed, the investigators consolidated them into a combined rapid analysis summary report for each FG, unifying themes and reconciling discrepancies by consensus through discussion. The summary reports for each FG were then transferred into a matrix in which each row was a participant quote and each column was a domain. From this matrix, investigators identified the underlying themes and subthemes between the 2 FGs.

## Results

### Overview

In total, 8 physical therapists (n=5, 63% men and n=3, 38% women), with a mean age of 47.5 (SD 5.6) years participated in this study. Participants reported obtaining PT degrees ranging from a bachelor’s degree to a Doctorate in Physical Therapy and had, on average, 20 (SD 8.5) years of clinical practice experience, and most reported practicing in an outpatient orthopedic setting. Of the 8 participants, 5 (63%) reported having advanced doctoral degrees (3/5, 60% PhD; 2/5, 40% EdD).

The qualitative results from the FGs were organized using the TAM for the acceptance of Motion Tape [38,40-42]. Data were organized based on the 3 main domains relevant to user acceptance (perceived wearability, perceived usefulness, and perceived ease of use) and 21 subthemes (Textbox 2). Subthemes were further designated using *positive*, *negative*, and *neutral* valences. Positive valence indicates that the FG participants perceived the Motion Tape attribute as positive. Negative valence indicates that the FG participants perceived the attribute as negative. Neutral valence indicates that the FG participants perceived the attribute as neither positive nor negative.

**Themes (n=3), subthemes (n=21), and valences of user acceptance of Motion Tape.**

**Theme 1: perceived wearability**

- Positive
  - Motion Tape has a small, universal fit.
  - The feeling of Motion Tape on the skin would decrease over time.
- Negative
  - Patients may feel Motion Tape’s wires snagging or sensors rubbing on clothes.
  - Motion Tape does not consider people with skin sensitivities.
- Neutral
  - Motion Tape adheres for 3-4 days but may adhere less owing to external factors.
  - The feeling of Motion Tape being removed depends on the physical therapist.

**Theme 2: perceived usefulness**

- Positive
  - Motion Tape could increase specificity of physical therapy management of low back pain (LBP).
  - Motion Tape could be effective for the diagnosis, management, and monitoring of low back pain (LBP).
  - The feeling of Motion Tape and the awareness of Motion Tape monitoring would increase adherence to a home exercise program.
  - Motion Tape would be beneficial in telerehabilitation and hybrid sessions.
  - Motion Tape could increase the physical therapist’s awareness of the pain source.
- Negative
  - Motion Tape brings legal concerns with data responsibility.
  - Motion Tape’s reliability could be affected by external factors.
- Neutral
  - Motion Tape could increase the efficiency of assessments, but set up could take more time.

**Theme 3: perceived ease of use**

- Positive
  - Motion Tape would be easy for a physical therapist to apply.
- Negative
  - Motion Tape has a lot of data to sift through.
  - Motion Tape data are hard to interpret in their current state.
  - The self-application of Motion Tape would be difficult.
  - Motion Tape is designed for single use.
- Neutral
  - The prescription of Motion Tape is subjective to many factors.
  - The user interface would dictate how much knowledge would be needed to use Motion Tape.

### Domain 1: Perceived Wearability

Regarding perceived *wearability*, all physical therapists were familiar with commercially available kinesiology tape. Thus, their thoughts about perceived wearability reflected their experience with kinesiology tape. For example, the physical therapists expected Motion Tape to last about 3 to 4 days. A physical therapist mentioned the following:

Oh, I’ve used the K-Tape for four days before it started peeling off. Sometimes it lasts more than five days actually. Three to four days I think is average.

FG1

However, some physical therapists clarified that the *longevity* of Motion Tape’s adhesion depends on several factors. For example, 2 of the physical therapists expressed the following:

How secure it is depends on a lot of factors, like moisture on the skin. It depends on not just moisture, but how clean your skin is and how much hair is on the skin.

FG2

Some of them, specifically on the low back, tend to have more oily skin, and that depreciates the life of the tape.

FG1

Regarding the *fit* aspect of wearability, physical therapists also believed that Motion Tape’s size was sufficiently small to be universal to the wearer and the placement location. They expressed the following:

In my experience with tapes like this, it fits most of the clientele that I’ve worked with, both inpatient and long-term post-acute.

FG1

If it was that little strip, I think it would be great to use anywhere.

FG2

Regarding the *feel* aspect of wearability, generally, physical therapists felt that patients would feel Motion Tape at first when applied but would become less aware over time until the tape starts to peel off:

They’d know that they’re there, and they’d probably become less aware of over time.

FG1

However, physical therapists generally felt that with Motion Tape’s current design, patients would *feel* the wires snagging or the sensors rubbing on clothing. A physical therapist explained it as follows:

So contraptions with wires will always have that uncomfortable feeling. Always. But if you go the wireless route, then probably after two days, the patient will be more comfortable until the tape starts peeling off. However, what I’m wearing right now, something that goes above my PSIS, if I go to the bathroom or do something, I’m going to, it’s gonna move around, it might get pulled on it by my clothes.

FG1

When removing Motion Tape, physical therapists said that patient feelings about the removal process would be quite variable. Some physical therapists felt that it was subjective to how the therapist removed Motion Tape and how much hair or oil the individual has on their skin. A physical therapist explained it as follows:

I’m just thinking of whoever is taking it off. You know, like, it depends on you, like, some people just rip. And some people are just gentle. So subjective. So it depends on the training of the therapist and concern if they’re empathetic to our patients.

FG1

The physical therapists mentioned some wearability concerns during the FGs. A concern was about how patients with skin sensitivities would be able to use Motion Tape. A physical therapist asked the following:

For those with skin allergy. Can you put an under wrap under this?

FG1

### Domain 2: Perceived Usefulness

Physical therapists expressed mixed feelings about whether Motion Tape would increase their efficiency with assessments of lumbar spine posture and movement. Some expressed that if all they had to do was apply the tape, then there would be increased efficiency:

If it’s easy to objectively document, by understanding the graph, I think it’s a night and day difference versus getting into the goniometers and doing manual assessment. Instead, you put on the tape, ask the patient to rotate their trunk, lean forward, reach forward, extend their back. And then if I have it digitally by email or direct messaging, it would save a bunch of time.

FG1

However, others felt that it would reduce efficiency. A physical therapist explained the following:

Regarding the speed of assessment, I would be a little doubtful. I think by the time that you took this and you put it on the patient, you hooked up all the wires to it, you did the calibration, if you need to do a calibration, it might take just as long as doing an assessment. I would have concerns around the accuracy of this, to give you a number, an accurate range of motion, particularly for things like rotation. But if the data was convincing that everyone, if it was validated for everyone that gave you an accurate number, I think it could improve the quality of assessment.

FG1

A physical therapist felt that for the in-clinic assessments, Motion Tape would improve specificity:

I don’t feel like it [Motion Tape] would improve speed, it would improve specificity.

FG2

Physical therapists also mentioned that they could envision Motion Tape as a useful tool for self-management and remote monitoring when used in combination with in-clinic PT. A physical therapist mentioned that the ability to monitor patients outside the clinic would be very meaningful:

That’s the best place to actually observe them, their normal environment. If they’re in therapy, they’re being observed, coached, cued by a skilled clinician. Their performance is definitely going to be different. So if they’re at home, and we’re able to monitor them at home, I think the treatment will be more, and your adjustment and progression will be more meaningful.

FG1

Some physical therapists suggested that having patients wear these sensors would increase their awareness of being monitored and thus increase engagement with and adherence to the home exercise program:

I think that what it has to offer is improving...adherence with our programs. I think that’s your potential.

FG1

When you tell someone, I’m looking at your posture right now, you change [gesturing to posture]. If they think you are watching, they’ll do better.

FG1

Physical therapists expected patients to have a phenomenal experience with Motion Tape when used in a hybrid setting:

I think to his point that if it’s applied properly in the clinics, it’s hybridized, and you can take a call, and there’s no technical involvement on the patient side, and all they do is open up the app, they’d have this really phenomenal experience.

FG2

Specifically, several physical therapists expressed that Motion Tape would help with the identification of postures and movements in free-living environments that provoke pain, allowing for more meaningful interventions:

I think for it to be very useful. It would have to compare with the app where you’ve got user input as to what’s going on...where he’s got these flags and the data that was pain here, pain here, pain here, and you can look, you know, to the periods of time before that.

FG2

Some physical therapists did have some concerns about the usefulness of Motion Tape. A physical therapist expressed legal concerns regarding data responsibility:

As long as you collect data, someone’s then responsible for it. So who’s going to look at it? What’s the liability then that person takes on by having that information?...if something goes wrong, and the therapist hasn’t looked at the data, I’d like to know, are they liable?

FG1

Another concern was knowing what external factors affect Motion Tape’s signal and data reliability, mentioning that the use of Motion Tape in practice was “gonna depend on the reliability of the data” (FG2).

Several physical therapists felt that there were a variety of variables that might affect the reliability of the signal or data. They expressed the following:

And what other factors affect them, the sensors, as far as humidity, water, other environmental factors that might affect it? You know, what if they have a compression garment around the trunk, for example, does that affect the sensors?

FG2

Whether, getting it wet and getting so some things on it changes the conductivity, and therefore the calibration over time.

FG2

You get variability in the readings based on amount of tension that people put on it when they applied it.

FG2

Whether that’s different from person to person because of different makeups morphology.

FG1

### Domain 3: Perceived Ease of Use

Physical therapists felt that it would be easy to apply Motion Tape, given their background knowledge in human anatomy. A physical therapist stated the following:

You would need to know basic clinical knowledge of the application for where to look for the muscles, you know, right. So, they need to be clinician to have knowledge of the body.

FG2

When asked whether they would feel comfortable using Motion Tape with their patients, there were mixed responses among physical therapists. Some mentioned that it would depend on “cost and buy-in” (FG2) or how it was going to be “incorporated into the plan of care” (FG1). A physical therapist even explained the variability as follows:

Depends on the situation, honestly. I mean, I have some families that I’ll show them how to do the application. And I’ll see them three weeks later, and they’ve reapplied four times and done it great. And then I’ve seen others that I’m like, “Oh, no! This is nope.”

FG2

There were also several concerns about the ease of use. Some physical therapists felt that they would have challenges with ease of use, specifically regarding interpretation of the data:

I think in its current form, easy to apply. Hard to interpret.

FG1

It depends on the interface and how much it interprets the points. The tape will be easy, but it’s all the other pieces.

FG2

Additional concerns about the ease of use included that the amount of data presented was excessive and the type of data displayed was difficult to interpret. The physical therapists expressed a desire to see the range of motion displayed in degrees rather than resistance in ohms:

I think I’m probably realistically just correlated with what they report has been painful. Because I don’t know that I’ve ever been so interested in all of that. Like, it might be too much data. For a patient, like I don’t necessarily need to know their range of motion during every single activity, I need to know when it is relevant to them. And when it is impacting whatever condition they’re here for.

FG1

And again, I think for a clinician, it’s going to have to be meaningful data. It’s gonna have be Range of motion data not ohms.

FG1

So then, conceivably, would it be helpful instead of giving you normalized strain,...if they could interpret it, would convert this over to degrees of rotation and flexibility?

FG2

If you could get range of motion kind of information, I think that would be great.

FG2

Another concern was about how challenging the self-application would be for patients:

How are people actually going to apply this on their own, someone that doesn’t know how?

FG2

Finally, another concern was that Motion Tape is a single-use product. A physical therapist explained the challenge of a single-use product as follows:

Okay, now how about waste? So it’s like a single use thing? Now I’m gonna throw in a whole planet into this is single usage. Or can you reapply?

FG2

### Future Recommendations

Future recommendations from the physical therapists were organized into 3 categories (Textbox 3): data, physical features, and app features.

**Themes (n=3) and subthemes of future recommendations for Motion Tape.**

**Theme 1: Data recommendations**

- Motion Tape data should be easy to read at a glance.
- Motion tape data should account for differing patient morphology.
- Physical therapists should be aware of factors that affect Motion Tape data.

**Theme 2: Physical feature recommendations**

- Motion Tape should be made wireless or with removable wires.
- Motion Tape should be reusable.
- Motion Tape should be customizable in length.

**Theme 3: App feature recommendations**

- Motion Tape app should include BMI input.
- Motion Tape app should include input for a patient’s change in activity.
- Motion Tape app should allow flagging events.
- Motion Tape app should include comparative data.

Regarding the data *recommendations*, physical therapists expressed that data should be summarized in the form of an at-a-glance graph with 1 overall meaningful number, reflecting the range of motion. They would also like to know how the data change from person to person owing to morphology and how external factors (water, application stretch variability, and skin movement) affect the data. Additional data that would be useful for their job included comparative data, graphs with a color scale, and information about muscle activation. Participants in an FG expressed the following:

Take a baseline and have them rotate from that position and determined by the volume strain, whether they are tension either degrees, or even if it’s yellow, green, yellow, red, like if they’re moving within if they can’t pinpoint it specifically, but you know, within a range, would that be helpful?

FG2

I think even just having comparative data would be helpful, right? Because, you know, I keep telling my students, “Don’t tell me, ‘I want to increase range 10 degrees.’” Because that doesn’t tell me, “Can they walk?” Right? But, “Are they doing better now than they were doing when we started?” That's useful. So even if we get baseline data that could be translated into amount of motion and then follow up data that says, “hey, it’s more, it’s more fluid, it’s better, it’s whatever.” I think that can be really useful. Now I know the payers are gonna want, how much rotation did you get? How much lateral flexion did you get?

FG2

And I think beyond the range of motion, I work in neuro. I think just like muscle activation would be interesting, you know, like, how much activation did you get today, for example, versus six weeks ago, post stroke or, you know, spinal cord or something? I think that would be really interesting just to see the firing muscle activation. And on the flip side, and I don’t know if that’s possible, but looking at specificity. Could that be something to monitor changes in specimen specificity? Post- X Y & Z intervention, right? That could also be interesting. So it’s not really about range of motion, we’re also activity known as firing or not?

FG2

Regarding *physical feature* recommendations, physical therapists wanted a way to mitigate the wires, either by moving to a wireless system or making them removable. Physical therapists were also concerned about the limited stretchability of the short pieces of tape, as it would not be long enough in length for typical kinesiology tape use, and recommended making the length customizable to the physical therapists’ needs. Physical therapists were also concerned about Motion Tape’s single-use design and were curious about whether it could be reusable to reduce waste:

Again, I’m thinking like, in the future, no wires, you’ve got a strip of graphene that you could customize length to, with those couple millimeters around the edge. And if we wanted a whole length, we cut whole lengths. And if we want segments, we can cut segments. And it feeds the data to the app somehow tailor it to someone’s body.

FG2

So you can imagine that maybe something like this could be a roll of tape. Yeah, the width of duct tape. And there’s actually two pieces on this roll. There’s one section, that’s the conductive piece, that you can cut it to length, and then next to it there are maybe there’s a wire section, that’s conductive tape that you can pull off and put on the ends of whatever you choose. So you get one roll of tape. And then one of them is the is the graphene is the other piece that you tear off to the appropriate length is the conductive tape that connects it to the box. And then it’s a solution, you can customize length and you have your conductive piece and then your graphene.

FG2

Regarding future *app feature* recommendations, physical therapists expressed a need for the capability to input factors such as BMI, activity changes, “flags” for events, and changes in pain to help label, compare, or contextualize the data.

## Discussion

### Overview

There is a gap in the research between rehabilitation device development and evaluation of clinicians’ acceptance of such devices. Most existing studies have considered patient or user satisfaction [47,48], whereas others that consider the clinician’s perspective have not specifically evaluated sensors for measuring spine posture and movement [49,50]. In this study, several themes relating to physical therapists’ perspectives about Motion Tape’s wearability, usefulness, and ease of use for a low back use case were identified.

### Domain 1: Perceived Wearability

One of the most common challenges for wearable sensors is ensuring that they are unobtrusive to the wearer’s natural movement and environment [39]. The small form and fit of Motion Tape was considered by physical therapists to be ideal for a wearable sensor. However, similar to previous studies, the wires in the current design were considered to be not ideal [37]. Studies have shown that wireless technologies tend to be more widely used in many fields, especially in the field of wearable devices for health care [51]. Thus, a future iteration of Motion Tape without wires would be considered optimal. On the basis of feedback obtained from physical therapists, wearability for people with skin sensitivities also should be considered. Previous studies have shown that skin irritation is the most common concern when using kinesiology tape for extended periods of time [52,53]. Thus, future studies should explore whether a medium or substrate can be used under Motion Tape to mitigate skin irritation, possibly as an extension of recent research that integrated Motion Tape with elastic fabric for respiration monitoring [54].

### Domain 2: Perceived Usefulness

There were mixed feelings among physical therapist participants about how efficient Motion Tape would be in the clinic. Overall, most physical therapists felt that Motion Tape would increase the specificity of their assessments, a characteristic that has been shown to be beneficial for LBP diagnosis and treatment [55]. Furthermore, Motion Tape’s ability to monitor the patient’s movements remotely was considered beneficial, as this feature may increase adherence to home exercise programs, which is an important component of effective treatment for LBP [56,57].

### Domain 3: Perceived Ease of Use

On the basis of physical therapists’ perspectives, Motion Tape would be easy to apply, but data would be difficult to interpret. Creating a device that is easy to use and understand is crucial because it predicts consumer use behavior [38,41]. Recommendations included presenting the data in units that physical therapists are more familiar with (ie, degrees of range of motion) and creating an app that requires minimal time for the physical therapists to use. These changes may promote increased device use and acceptance in PT.

### Future Recommendations

On the basis of clinician feedback, Motion Tape appears to be a promising new technology that could be used for monitoring lumbar spine posture and movement in the management of patients with LBP. Future device development will be needed to address clinician recommendations obtained from this study in the domains of wearability and ease of use. In addition, future studies will be needed to validate Motion Tape in laboratory, clinical, and free-living environments and to investigate patient acceptance of Motion Tape.

### Limitations

A limitation of this study is that participants were physical therapists who were part of a Technology Special Interest Group and are likely to be more receptive to using technology in practice. Thus, this study’s results regarding Motion Tape’s acceptability may be biased in favor of Motion Tape’s ease of use, usefulness, and wearability. Future studies should also assess the acceptability of Motion Tape for clinicians who do not regularly use technology in their practice. Another limitation is that the physical therapists were not presented with active samples of Motion Tape with live data streams in the app. Instead, participants were given inactive samples of Motion Tape and presented with a poster with examples of app data streams. Future studies should provide an opportunity for physical therapists to apply Motion Tape to a person and use it with the app interface. Finally, there was a potential for investigator bias in the interpretation of the results, as several investigators of this study are actively working on the development of this device. However, 2 of the 3 investigators who conducted data analysis were outside the primary research team.

### Conclusions

Physical therapists expressed overall acceptance of Motion Tape for its potential to monitor and assess low back posture and movement, both within and outside clinical settings. Physical therapist participants expressed that Motion Tape would be a valuable tool for personalized treatment of LBP but highlighted several future improvements needed for Motion Tape to be used in practice.

## Appendix

Multimedia Appendix 1 Focus group guide.

## Abbreviations

APTA: American Physical Therapy Association
FG: focus group
HIPAA: Health Insurance Portability and Accountability Act
IMU: inertial measurement unit
LBP: low back pain
PT: physical therapy
RQA: rapid qualitative analysis
TAM: Technology Acceptance Model
