# Supplementary material for: Physical Therapists’ Acceptance of a Wearable, Fabric-Based Sensor System (Motion Tape) for Use in Clinical Practice: Qualitative Focus Group Study
Source: JMIR Hum Factors. 2024 Feb 29;11:e55246. doi: 10.2196/55246 (PMC10940997; doi:10.2196/55246)
Supplement: Multimedia Appendix 1 [file humanfactors_v11i1e55246_app1.docx]

Last Updated: 2/1/23, AL

**INTERVIEW GUIDE**

**Kinesiology Tape Sensor Feedback Survey**

**Introductions**

**Interviewer:**

*Good morning/afternoon. My name is _______________________________. The purpose of this interview is to obtain your feedback about the Kinesiology Tape (“K-Tape”) Sensors.*

*We have asked you to take part in this interview because you are a Physical Therapist or Clinician by profession and could help us get a better understanding about how people would react to these K-Tape Sensors. We want to learn from you. Please feel free to give us your honest feedback about the K-Tape Sensors, even if you do not like them. Your ideas and opinions are important as we continue to improve these sensors for use in the community.*

*Our interview today will last approximately 1 hour. It is informal, and there are no right or wrong answers. Your participation is voluntary. If you feel like you need a break or want to stop, please let me know. The information that you tell me today will be kept private. A final report will be prepared using the information that you give me, but your name and other identifying information will not be used. We hope that you feel comfortable to speak freely.*

*For this interview, I have several questions to ask you. We know that your time is important, so we want to be sure to keep you only for the time that we promised. To make that happen, I may ask you to finish your thought or sentence and then move on to another topic or question.*

*I will ask if I may tape record our conversation so that I can pay more attention to you and what you have to say instead of trying to write everything down. We will transcribe our interview from this tape. Is it okay for me to begin recording now?*

*[If permission given, begin recording.]*

**Date: ________________________________ Participant #: _______**

**Say into audio recorder: This is (*Name of Researcher*). Today is (*date*), and I am interviewing**

**participant number (*Participant Number*).**

**Thank you so much for taking part in the interview.  Are you ready to begin?**

1. Pass out K-Tape sensor to the PT/Clinician to observe.
2. Introduction:

*Adherence to PT at-home exercises is important for improving patient outcomes. Remote monitoring of exercises has the potential improve adherence to these exercises for optimal treatment and recovery.*

*Before you, are K-Tape Sensors. We are working with a collaborative team to develop these K-Tape sensors for measuring low back movement and muscle activity. These sensors would be placed on the patient’s back and provide feedback to both the patient and the clinician about the patient’s movements and muscle activity. On this posterboard, is example pilot data that the device would be able to give you as a clinician.*

1. First impression question:
   1. *What is your first impression of the K-Tape Sensors?*
   2. *What from this set of data would be more useful to you, if any?* [TAM_U6]
      1. *Why?*
2. Wearability: an added variable to the TAM [KEY: W, Factors of Analysis: (1) adhesion, (2) fit, (3) feel, (4) application/prescribing]

Give the PT/clinician the opportunity to put sensors on a research staff member

*I would now like you to answer the following questions regarding the wearability of these K-Tape sensors. Wearability is the degree to which you believe the sensors fit well and are comfortable on your patient.*

- 1. *How secure do you think the K-Tape adhesive will be?* [W_1]
     1. *PROBE*: *What if it is worn for an entire day/several days?*
  2. *To what degree do you think these sensors would anatomically fit on your patients’ backs?* [W_2]
  3. *To what degree do you think your patients would feel the sensors on their back?* [W_3]
     1. *PROBE: How well do you think the K-Tape would feel under the patient’s normal clothes while they went about their normal routine?*
     2. *PROBE: Do you think their level of awareness would affect their range of movement? Why or why not? [W_3]*
     3. *PROBE: Do you think their level of awareness would affect their performance of exercises? Why or why not?* [W_3]
  4. *How do you predict the K-Tape Sensors would feel when being removed?* [W_3]
  5. *How comfortable would you feel prescribing K-Tape to a patient to monitor their movements at home?* [W_4]

1. Perceived usefulness: [KEY: TAM_U, Factors of Analysis: (1) work more quickly, (3) increase productivity, (4) effectiveness, (5) makes job easier, (6) useful]

*I would now like you to answer the following questions regarding the perceived usefulness of these K-Tape sensors. Perceived usefulness is the degree to which you believe that using these sensors would enhance your job performance.*

- 1. *To what degree would the usage of K-Tape sensors affect how quickly you can assess your patient’s Posture, movement, and/or exercise performance?* [TAM_U1,3]
  2. *How effective do you think the K-Tape sensors will be to capture valid data on your patients in the* ***clinic****?* [TAM_U4]
  3. *How effective do you think the K-Tape will be to capture valid data on your patients in their* ***daily routine and normal environment****?*
  4. *To what degree would the usage of K-Tape sensors affect the level of difficulty of your job as a clinician/PT? [TAM_U5]*
     1. *PROBE: How would the usage of these K-Tape sensors affect your productivity as a clinician/PT?* [TAM_U3,1]
     2. *PROBE: Would it make it easier or harder? Why?*
  5. *What features, if any, would make the K-Tape more useful to you?* [TAM_U6]
     1. *PROBE*: *Why or why not?*

1. Perceived ease of use: [TAM_EU, Factors of Analysis: (1) easy to learn, (2) clear and understandable, (3) easy to use]

*I would now like you to answer the following questions regarding the perceived ease of use of these K-Tape sensors. Perceived ease of use is the degree to which you believe using these sensors would be free of effort.*

- 1. *How easy do you think it would be to learn how to use K-Tape?* [TAM_EU1]
  2. *What level of knowledge do you think a clinician/PT would need to use the K-Tape?* [TAM_EU3]
     1. *PROBE: Do you think any specialized training would be required? Why or why not?*
     2. *PROBE: Would it appropriate for a novice/expert/clinician?*
  3. *How easy/difficult do you think it would be for a clinician/PT to apply the K-Tape to the patient's back?* [TAM_EU6]
  4. *What features, if any, would make the K-Tape easier for you to use?*
     1. *PROBE*: *Why or Why not?* [TAM_EU6]

1. Concluding questions:
   1. *What kind of benefits, if any, do you think K-Tape can offer?*
      1. *PROBE: Why?*
   2. *What kind of limitations, if any, do you think K-Tape presents?*
      1. *PROBE: Do you think wearing the K-Tape would pose any concerns?*
         1. *PROBE: Why or why not?*
      2. *PROBE: What, if anything, would you like to see improved about the K-Tape Sensors?*
         1. *PROBE: Why?*
   3. *If this sensor was offered in pair with remote physical therapy (by phone or video), do you predict patients would utilize PT remote services more often?*
      1. *PROBE: Why or why not?*

More Response Probing

1. *How have you reached this answer?*
2. *How did you arrive to that answer?*
3. *What made you say that?*
4. *Is there anything else you would like to add?*
5. Restate their answer. For example, *“So you believe you are able to complete your normal daily routine, despite wearing the K-Tape Sensors?”*
6. *Can you take me through the steps of how you came to that answer?*
7. When done with the interview, say to the participant*:*

*“Thank you so much for taking part in the interview. I am going to stop recording now”.*
